# Prevalence and contributing factors of malocclusion in Zhuang children aged 7–8 years in southern China

**DOI:** 10.3389/fped.2024.1308039

**Published:** 2024-01-15

**Authors:** Wenjia Mai, Lijuan Xiao, Shaoyong Chen, Shuang Chen, Andi Li, Tingting Zhang, Haoyu He, Xiaojuan Zeng

**Affiliations:** ^1^ Department of Orthodontics, College & Hospital of Stomatology, Guangxi Medical University, Nanning, Guangxi, China; ^2^ Department of Dental Public Health, College & Hospital of Stomatology, Guangxi Medical University, Nanning, Guangxi, China

**Keywords:** cross-sectional study, malocclusion, prevalence, mixed dentition, Zhuang children

## Abstract

**Introduction:**

Malocclusion, a common oral health problem in children, is associated with several contributing factors. This study aimed to investigate the prevalence of mixed dentition stage malocclusion and its contributing factors in Chinese Zhuang children aged 7–8 years.

**Methods:**

Overall, 2,281 Zhuang children, about 7–8 years old, were randomly selected using a stratified whole-cluster sampling method from schools in counties in Northwestern Guangxi, China. The children were examined on-site for malocclusion and caries by trained dentists, and basic data on the children were collected using questionnaires, including age, sex, parental education, parental accompaniment, and children's knowledge of malocclusion and treatment needs. Data were analyzed using the chi-square test and logistic regression analysis.

**Results:**

The total prevalence of malocclusion in Zhuang children aged 7–8 years was 58.5%, with the highest prevalence of anterior crossbite tendency, and the prevalence of anterior crossbite and anterior edge-to-edge occlusion was 15.1% and 7.7%, respectively. This was followed by an anterior increased overjet of 13.3% and an inter-incisor spacing of 10.3%. The lowest prevalence was 2.7% for anterior open bite. Sex, parental accompaniment, parental education, and decayed, missing, and filled teeth of the first primary molar were factors that contributed to malocclusion in Zhuang children.

**Conclusion:**

Malocclusion is a common oral problem among Zhuang children. Therefore, more attention must be paid to the intervention and prevention of malocclusion. The impact factors should be controlled as early as possible.

## 1 Introduction

Malocclusion is a variety of deformities caused by irregularities in the relationship between the maxillary and mandibular teeth, dental arches, and hard and soft tissues of the craniomaxillofacial region. Malocclusion is regarded as one of the three primary oral disorders that impact oral functionality, facial appearance, social engagement, as well as the physical and mental well-being of individuals (1).

When the first permanent molars in schoolchildren, aged 7–8 years old, establish occlusal contact, the maxillary and mandibular anterior begin to erupt. This stage is considered critical for the later establishment of a harmonious and stable occlusal relationship and is also the starting stage for early intervention in correcting malocclusion during the mixed dentition period (2). Studies have shown that malocclusion during the mixed dentition phase can lead to malocclusion of the permanent dentition if early intervention is not performed. In addition, the adverse consequences of malocclusion during mixed dentition, even at an early age, may negatively impact children's emotions and social aspects (3).

A systematic review and meta-analysis found that the pooled national prevalence of malocclusion in Chinese schoolchildren from 1991 to 2018 was 47.92%. However, the prevalence, which ranged from 30.07% to 75.43%, exhibited significant variation across different regions of China (4). In addition, many studies have indicated a high occurrence of malocclusion in permanent teeth, with reported rates varying between 10.0% and 97.0% (5, 6). This variation may be ascribed to variances in territory, customs, race, economic conditions, survey standards, and indicators (7). To date, epidemiological descriptions of mixed dentition malocclusion worldwide remain scarce, and information on the prevalence of malocclusion in children with mixed dentition in the Chinese population, especially in ethnic groups, is limited.

The Zhuang is the largest minority ethnic group in China, second only to the Han, and its population is mainly located in the Guangxi Zhuang Autonomous Region of southern China. Counties in western Guangxi are the main sources of Zhuang. The high prevalence of caries, low oral health awareness, low socioeconomic status, and low parental accompaniment in the Zhuang region may have influenced the occurrence of malocclusion in children residing in this region (8). Nevertheless, there is insufficient data regarding the frequency of malocclusion among children of the Zhuang ethnicity in China. The objective of this research was to investigate the prevalence of malocclusion during the early mixed dentition period and its contributing factors in Guangxi Zhuang children. To identify the elements that influence malocclusion during this period and implement strategies for prevention and early intervention can decrease the prevalence and severity of malocclusion in the future (9, 10).

## 2 Materials and methods

### 2.1 Sample selection

This study was conducted in Guangxi Province, South China, in 2022. Four counties, Jingxi, Long'an, Tiandeng, and Dahua, were randomly selected from western Guangxi province. All participants under investigation from these counties were 7–8 years old and belonged to the Zhuang ethnic group, which is China's largest minority residing in Guangxi.

The sample size was calculated using the sample size formula $N=\mathrm{deff}\frac{\mu_{\alpha /2}^{2}p(1\text{-}p)}{\sigma^{2}}$. Owing to the lack of data on the prevalence of malocclusion in children aged 7–8 years in this region, the estimated rate p was set at 50%, the relative permitted error *δ* of the overall rate *p* was set at 5%, the test level was bilateral *α* = 0. 05, *μα*/2 = 1.96, and the efficiency of the sampling design was deff = 4.5, which was calculated as a sample size of 1,729 children. Taking into account a non-response rate of 20%, the sample size needed was 2,162. The total number of people in the four counties surveyed was 2,281, which was larger than the required sample size.

This study excluded children who had previously received or were currently receiving orthodontic treatment, as well as those with systemic diseases or obvious craniofacial deformities like ectodermal dysplasia, Down syndrome, cleft lip, and/or palate. Exclusion also applied to children who declined to take part in the assessment and those who were uncontactable or declined to give their consent to participate.

The Ethical Review Committee of Guangxi Medical University College of Stomatology granted approval for this study (No. 2022043). Consent forms were obtained from the parents or guardians of all children involved in this study, indicating their informed agreement.

### 2.2 Clinical examination

Malocclusion and caries in all participants were examined by trained and calibrated dentists using a Community Periodontal Index probe, plane mouth mirror, and measuring ruler under an artificial light source. The types of malocclusions, including upper anterior space, anterior overjet, anterior edge-to-edge occlusion, anterior crossbite, anterior overbite, anterior open bite, posterior transverse discrepancy, and Angle classification, were defined (Table 1). Additionally, the decayed, missing, and filled teeth (dmft/DMFT) scores of the primary canine, first primary molar, second permanent molar, and first permanent molar were recorded separately. The scores were statistically analyzed according to categorical information, categorized according to the corresponding tooth positions, and labeled as dmft/DMFT = 0, dmft/DMFT = 1, dmft/DMFT = 2, dmft/DMFT = 3, and dmft/DMFT = 4. The World Health Organization diagnostic criteria for dental caries were used to evaluate the dental caries status.

**Table 1:** Types of malocclusion and related diagnostic criteria.

Types	Diagnosis criteria	
Upper anterior space	Normal (<=2) Excess (>2)	Recorded the space between maxillary mesial incisors.
Anterior overjet	Normal (>0, <=3)	Recorded the distance from the incisal edge of the most labial maxillary incisors to the most labial surface of the corresponding mandibular incisors.
	Excess (>3)	
	Edge-to-edge occlusion (=0)	
	anterior crossbite (<0)	
Anterior overbite	Normal (=<1/3 lower incisors)	Recorded the vertical overlap of the upper and lower incisors.
	Excess (>1/3 lower incisors)	
Anterior open bite	Absent Present (>0)	Recorded the vertically separated distance between the upper and lower incisors.
Posterior transverse discrepancy	Absent Present	Recorded the molars with crossbite or scissor-bite relationship.
Angle classification	Angle Class I Angle Class II Angle III	Recorded the molars’ relationship between the mesiobuccal cusp of the maxillary first molar and the buccal groove of the mandibular first molar. When the first molars were not present in occlusion, the second primary molars were used.

To guarantee the accuracy and dependability of data collection, a total of eight examiners underwent training and calibration. Before conducting the formal investigation, we examined 108 children aged 7–8 years. 8 examiners, each 2 as a group, with a group of 2 senior doctors as the gold standard, and the results of each group were tested for inter-examiner reliability with the gold standard by Kappa values. 1 week later, we re-examined these 108 children as a way of calculating intra-examiner reliability. The inter-examiner reliability had Kappa values ranging from 0.84 to 0.91, while the intra-examiner reliability had Kappa values ranging from 0.86 to 0.95.

Radiographic examinations were not performed. At the end of the oral examination, a written notification was dispatched to the child's parents or guardians, informing them of the necessity for the child to receive treatment.

The questionnaire was designed in Chinese to collect data on sociodemographic variables, including age, sex, parental education (lower than college degree/ college degree and higher), and parental accompaniment (parents/only father/only mother/no parents). Parental accompaniment was counted based on whether the parents accompanied the children home in the last 6 months. Additionally, a questionnaire was employed to gather information regarding the children's knowledge of orthodontics (known/unknown) and their self-need for orthodontics (yes/no/unknown). Before starting the formal questionnaire, we conducted a pre-survey of 108 children aged 7–8 in Chinese. The trained examiners explained all the questions of the questionnaire to the children in detail, and all the questions raised by the children were satisfactorily resolved. Eventually, all children completed the questionnaire under the supervision and assistance of the examiners. To retest the reliability of the questionnaire, these 108 children were re-examined a week later. The Kappa values were all higher than 0.88.

### 2.3 Statistical analysis

The Statistical Package for the Social Sciences software (IBM SPSS Statistics, version 25) was utilized for conducting the statistical analyses. The prevalence of malocclusion (%) and categorical variables were calculated. Proportions were compared using the chi-square test. Statistically significant differences were deemed when the *P*-value was less than 0.05. This study utilized binary logistic regression to examine the impact of the independent factors on malocclusion in children. In terms of statistics, various factors were included in a logistic model, and those that did not demonstrate a significant correlation were subsequently eliminated using a stepwise approach.

## 3 Results

A total of 2,344 Zhuang children from four counties in Guangxi (Tiandeng, Long'an, Dahua, and Jinxi) were asked to undergo a dental examination. Out of these, a total of 2,297 individuals consented to take part in the research, resulting in a response rate of 96.4%. Finally, 2,281 children were analyzed, excluding 16 with incomplete data.

### 3.1 Prevalence of malocclusion

Among the 2,281 children clinically examined, 947 (41.5%) had normal occlusion, whereas 1,334 had malocclusion, with a total malocclusion prevalence of 58.5%. Of the 1,334 children, the highest number of malocclusions was Angle Class I, with 607 children, with a prevalence of 26.6%, followed by Angle Class II, with 532 children, with a prevalence of 23.3%, and the lowest number of children with Angle Class III malocclusion was 195, with a prevalence of 8.5% (Table 2).

**Table 2:** Prevalence of different types of malocclusion based on demographic characteristics (Chi-square test).

	Number of participants	Total prevalence of malocclusion *n* (%)	*P*-value (x^2^)	Angle classification of malocclusion *n* (%)			Upper anterior space *n* (%)	Anterior overjet *n* (%)	Anterior overbite *n* (%)	Anterior crossbite tendency *n* (%)		Anterior open bite *n* (%)	Posterior transverse discrepancy *n* (%)
				Class I	Class II	Class III				Edge-to-edge	Crossbite		
Age			0.021 (5.287)										
7 years old	701	385 (54.9)		158 (22.5)	169 (24.1)	58 (8.3)	46 (6.6)	51 (7.3)	62 (8.8)	62 (8.8)	99 (14.1)	14 (2.0)	31 (4.4)
8 years old	1,580	949 (60.1)		449 (28.4)	363 (23.0)	137 (8.7)	189 (12.0)	253 (16.0)	89 (5.6)	113 (7.2)	245 (15.5)	47 (3.0)	93 (5.9)
Sex			0.009 (6.807)										
Female	1,020	566 (55.5)		254 (24.9)	222 (21.8)	90 (8.8)	88 (8.6)	121 (11.9)	56 (5.5)	87 (8.5)	148 (14.5)	31 (3.0)	68 (6.7)
Male	1,261	768 (60.9)		353 (28.0)	310 (24.6)	105 (8.3)	147 (11.7)	183 (14.5)	95 (7.5)	88 (7.0)	196 (15.5)	30 (2.4)	56 (4.4)
Total	2,281	1,334 (58.5)		607 (26.6)	532 (23.3)	195 (8.5)	235 (10.3)	304 (13.3)	151 (6)	175 (7.7)	344 (15.1)	61 (2.7)	124 (5.4)

The clinical classifications of the malocclusions are presented in Table 2. The prevalence of anterior crossbite tendency was the highest at 15.1% and 7.7% for anterior crossbite and anterior edge-to-edge occlusion, respectively. This was followed by an anterior increased overjet of 13.3% and an inter-incisor spacing of 10.3%. The lowest prevalence was 2.7% for anterior open bite.

### 3.2 Relationship between malocclusion and demographic characteristics

Table 2 displays the prevalence of malocclusion in children with various demographic characteristics. The findings indicated that the prevalence of malocclusion among children aged 8 was notably greater (60.1%) compared to children aged 7 (54.9%). Furthermore, the prevalence of males was considerably greater (60.9%) compared to females (55.5%). In addition, children who resided without their parents' company exhibited the greatest prevalence of malocclusion (67.3%, *P* < 0.001). By two-by-two comparison, it was found that the prevalence in the unaccompanied group was significantly higher than that in the only mother- and parent-accompanied groups; however, there was no statistically significant distinction with the group accompanied by only fathers. Additionally, a correlation existed between parental education and the prevalence of malocclusion. The prevalence of malocclusion in the children of parents with lower than a college education was significantly higher in both groups (65.2% and 65.3%) than in the group with a college education and higher (43.0% and 44.6%).

### 3.3 Relationship between malocclusion and dmft/DMFT

Table 3 presents the analysis of malocclusion according to different dmft/DMFT in children's primary canine teeth, first primary molars, second primary molars, and first molars. Except for the first molar, significant variations in the dmft scores were observed among the remaining teeth. The examination of dmft in primary canine teeth revealed that the dmft = 4 group had the highest prevalence of malocclusion (79.1%, *P* < 0.05), while the dmft = 0 group had the lowest prevalence of malocclusion at 56.4%. Interestingly, in the analysis of dmft scores of the first and second primary molars, it was found that the prevalence of malocclusion with dmft = 4 was the highest at 62.7% and 63.3%, whereas the prevalence of malocclusion with dmft = 0 was the lowest at 52.0% and 53.3%, respectively, and these variations were statistically significant. In addition, in the analysis of the DMFT scores of the first molars, the prevalence of malocclusion with DMFT = 4 was still the highest, but the disparity lacked statistical significance.

**Table 3:** Malocclusion in children with socio-demographic characteristics, dmft/DMFT, orthodontic perceptions and self-needs (*n* = 2,281).

	Normal *n* (%)	Malocclusions *n* (%)	*P*-value (x^2^)
Parental companionship			<0.001 (32.580)
Parents	468 (46.4)	540 (53.6)^b^	
Only father	68 (42.5)	92 (57.5)^a,b^	
Only mother	189 (43.5)	245 (56.5)^b^	
No parents	222 (32.7)	457 (67.3)^a^	
Father's education			<0.001 (97.962)
College degree and higher	393 (57.0)	296 (43.0)	
Lower than college degree	554 (34.8)	1,038 (65.2)	
Mother's education			<0.001 (88.786)
College degree and higher	416 (55.4)	335 (44.6)	
Lower than college degree	531 (34.7)	999 (65.3)	
Primary canine dmft			0.014 (12.429)
dmft = 0	656 (43.6)	847 (56.4)	
dmft = 1	105 (39.6)	160 (60.4)	
dmft = 2	97 (39.0)	152 (61.0)	
dmft = 3	46 (38.3)	74 (61.7)	
dmft = 4	43 (29.9)	101 (70.1)	
First primary molar dmft			0.008 (13.787)
dmft = 0	237 (48.0)	257 (52.0)	
dmft = 1	117 (43.2)	154 (56.8)	
dmft = 2	196 (40.4)	289 (59.6)	
dmft = 3	166 (40.3)	246 (59.7)	
dmft = 4	231 (37.3)	388 (62.7)	
Second primary molar dmft			0.017 (12.112)
dmft = 0	255 (46.7)	291 (53.3)	
dmft = 1	111 (42.4)	151 (57.6)	
dmft = 2	206 (41.4)	291 (58.6)	
dmft = 3	148 (41.5)	209 (58.5)	
dmft = 4	227 (36.7)	392 (63.3)	
First molar DMFT			0.713 (2.122)
DMFT = 0	822 (41.7)	1,149 (58.3)	
DMFT = 1	71 (41.5)	100 (58.5)	
DMFT = 2	38 (37.3)	64 (62.7)	
DMFT = 3	12 (50.0)	12 (50.0)	
DMFT = 4	4 (30.8)	9 (69.2)	
Orthodontic perceptions			0.136 (2.218)
Know	507 (43.0)	672 (57.0)	
Unknown	440 (39.9)	662 (60.1)	
Orthodontic self-needs			0.002 (12.318)
Yes	117 (36.1)	207 (63.9)^a^	
No	460 (45.4)	553 (54.6)^b^	
Unknown	370(39.2)	574(60.8)^a^	
Total	947(41.5)	1,334(58.5)	

^a,b^ two-by-two comparison in Chi-square test.

### 3.4 Relationship between malocclusion and orthodontic perceptions and self-needs

Regarding children's orthodontic self-needs, the chi-square analysis showed that the group of children who needed orthodontic treatment for their own malocclusion had the highest prevalence of malocclusion (63.9%, *P *< 0.05). The prevalence of malocclusion in the group of unknowns was 60.8%, which was significantly higher than the prevalence in the group of children who did not need orthodontic treatment (54.6%). Table 3 showed that there was no notable correlation between children's knowledge of orthodontics and the prevalence of malocclusion.

### 3.5 Binary logistic regression analysis of contributing factors and malocclusion

The prevalence of malocclusion in Zhuang children was influenced by sex, parental accompaniment, parental education, and dmft of the first primary molar, as indicated by the results of conditional binary logistic regression analysis. (*P* < 0.05, Table 4). Male children had a 1.271-fold increased risk of malocclusion compared to female children. Children who live without parental accompaniment have a 1.656 times greater risk of malocclusion compared to children who live with their parents. The risk of malocclusion in children living without parental accompaniment was 1.656 times higher than that in children living with parents. Whereas the prevalence of malocclusion in children accompanied by only their mothers or fathers was different from that in children accompanied by their parents, but the difference did not have statistically significant. In addition, the risk of malocclusion in children with fathers with lower than a college education was 1.840 times higher than in those with a college education and higher. The risk of malocclusion in children with mothers with lower than a college education was 1.595 times higher than in those with a college education and higher. Furthermore, children with first primary molar dmft ≥ 2 had a greater risk of developing malocclusion compared to those with first primary molar dmft = 0 (odds ratio 1.374, 1.389, and 1.559, respectively).

**Table 4:** Binary logistic regression analysis of contributing factors and malocclusion.

		B	standard error	*P*	Odds ratio(95%Cl)
Sex	Female				
	Male	0.240	0.089	0.007	1.271 (1.068–1.513)
Parental companionship	Parents			<0.001	
	Only father	0.134	0.177	0.448	1.144 (0.809–1.617)
	Only mother	0.138	0.12	0.249	1.148 (0.908–1.451)
	No parents	0.504	0.107	<0.001	1.656 (1.344–2.040)
Father's education	College degree and higher				
	Lower than college degree	0.610	0.117	<0.001	1.840 (1.464–2.313)
Mother's education	College degree and higher				
	Lower than college degree	0.467	0.114	<0.001	1.595 (1.275–1.996)
First primary molar dmft	dmft = 0			0.009	
	dmft = 1	0.180	0.157	0.253	1.197 (0.879–1.630)
	dmft = 2	0.318	0.133	0.017	1.374 (1.058–1.785)
	dmft = 3	0.329	0.140	0.019	1.389 (1.056–1.827)
	dmft = 4	0.444	0.127	<0.001	1.559 (1.215–1.999)
	Constant	−0.963	0.137	<0.001	0.382

## 4 Discussion

The prevalence of malocclusion varies according to region and ethnicity. This study analyzed 2,281 Zhuang children, approximately 7–8 years of age, and found that the overall prevalence of malocclusion was 58.5%. The prevalence was lower than the 71.21% reported in a national malocclusion prevalence survey conducted in China in 2000 (2). Nevertheless, the prevalence in this investigation surpassed the pooled prevalence of malocclusion in Chinese students over the last 30 years (47.92%) reported in a systematic review (4). However, the prevalence of malocclusion among 6–8-year-old children in mixed dentition public schools in southern Brazil is 69.1%, and a systematic review showed that the pooled prevalence of malocclusion among children in India is 66.7% (confidence interval 50.7–81.06) (11, 12). This may be related to the following reasons: First, the prevalence in different regions and ethnic groups may have certain differences; Zhuang children are mainly located in southern China; the Zhuang region has poor economic conditions; people have low awareness of oral health; and the prevalence of caries is high, which may influence the high prevalence in Zhuang children (8). In addition, the prevalence of crowded teeth was not counted in this study because the anterior teeth of children aged 7–8 years had just erupted, there was temporary malocclusion, and later, with the replacement of teeth and the growth of the jaw, the temporary misalignment could be corrected on its own; therefore, the prevalence of crowding was not statistically significant. In addition, in this survey, the highest incidence of Angle Class I malocclusion was 26.6%, with Angle Class II malocclusion (23.3%) and Angle Class III malocclusion (8.5%) following suit. These findings align with previous research conducted in various countries (1, 13–15).

The highest prevalence in the clinical classification of malocclusion was observed in the tendency for anterior crossbite, with anterior crossbite and anterior edge-to-edge occlusion having prevalence rates of 15.1% and 7.7%, respectively. According to the prevalence of Angle classification, it is estimated that the anterior crossbite during the mixed dentition phase of Zhuang children is mostly dentary or functional. In addition to genetic factors and bad oral habits, anterior crossbite is associated with tooth decay, premature primary tooth loss, and an irregular eruption sequence of permanent teeth.

In this study, the second most prevalent malocclusion observed was an anterior increased overjet (13.3%), which agrees with observations reported in Tanzania (16). In addition to different ethnicities and genetic factors, increased anterior overjet is also associated with bad oral habits, such as tongue thrust, digit sucking, lower lip biting, and mouth breathing (17, 18). When treating deep overjet in mixed dentition at an early stage, the following factors should be considered: (1) increased overjet affects children's appearance, which has an impact on their psychological health and makes them easy targets for ridicule by other students (19); (2) children with maxillary anterior protrusion have a higher risk of traumatic injuries to their upper anterior teeth (20); (3) increased overjet is more likely to cause an increase in the plaque index in the anterior tooth area and gingival inflammation (21); (4) early intervention in children with mandibular retrusion can improve the dimensions of their upper airway and reduce the potential risk of developing obstructive sleep apnea syndrome in the future (22).

This study also found that the prevalence of malocclusion was greater in male children than in females, which is in accordance with the results in Germany. Christopher et al. reported that the prevalence of deep cover and overlay in 9-year-old children in Germany was higher in males than in females (23). The growth spurts during puberty happen at 13 years old for males and 11 years old for females. Females grow faster than males by 2 years, and this can impact the occurrence of mixed dentition malocclusion. This is because certain temporary malocclusions like increased overjet, deep overbite, crowding, and anterior spacing are resolved as the jaws grow and permanent teeth come in (24).

The prevalence of malocclusion in children was linked to the educational background of their parents. Parents with lower than a college education had a considerably greater prevalence of malocclusion in their children than those with a college education and higher. The growth and development of children are also significantly influenced by parents. This study revealed that malocclusion was most common among children who lived without their parents, which was considerably higher compared to children who were accompanied by only mother and parent groups. However, the difference was not statistically significant when compared to the group accompanied by only father. This suggests that there is a connection between parental accompaniment and malocclusion in children, especially the mother's accompaniment, which has a significant impact on children's growth and progress. Binary logistic regression also suggested that children without parental companions have a 1.656 times greater risk of malocclusion compared to those with parental companions. Parents with higher educational levels have more ways to acquire oral healthcare knowledge and are more receptive to it. The presence of parents during the growth period of children is beneficial for the timely detection of oral problems and consultation, helps to cultivate proper oral habits, and reduces malocclusion caused by bad oral habits. A study in Sichuan showed that the lack of parental accompaniment increases the likelihood of left-behind children experiencing mental health issues, as well as their hesitancy to confide in others regarding their emotional distress (25). The increased prevalence of malocclusion in children could be attributed to these factors.

Significant lifestyle and dietary changes have occurred as a result of the swift growth of China's economy. The intake of large quantities of fine foods reduces the stimulation of the masticatory muscles and affects normal orofacial development and occlusal relationships, and the intake of large quantities of sugary foods has resulted in a growing incidence of tooth decay in the primary dentition of children each year (26, 27). In China, Guangxi is the primary region for sugar production, leading to a higher likelihood of local residents consuming sugary food items. Numerous candies, cookies, carbonated beverages, and sugar-laden foods are sold in numerous school cafeterias (28). Excessive consumption of sugar has been linked to the high occurrence of dental cavities in children residing in Guangxi, as indicated by previous research (27). This study found a correlation between the decay of primary canine teeth and primary molars and malocclusion in children. The decay of the first primary molars posed a potential danger for malocclusion in children, and children with a dmft ≥ 2 score had a higher risk of developing malocclusion compared to those with a dmft = 0 score (odds ratio values were 1.374, 1.389, and 1.559) in this study. Indian researchers have also reported similar findings, demonstrating a positive association between the severity of malocclusion and dmft /DMFT. Higher dmft /DMFT scores were found to indicate more severe malocclusion (29, 30). Decay or premature loss of primary teeth will affect masticatory function in children, and it is easy to develop bad chewing habits such as unilateral posterior teeth chewing and anterior teeth chewing. In addition, premature loss of primary teeth will lead to the movement of adjacent teeth to the missing teeth, which will affect the occlusal relationship in that area, simultaneously resulting in insufficient eruption space for the permanent teeth in that area. Therefore, the permanent teeth will not be able to erupt normally or erupt in an ectopic manner, ultimately affecting the occurrence of malocclusion (17, 31). However, this study can only conclude that dmft of the first primary molars poses a potential hazard for malocclusion in children. Further analysis of the causal relationship between dmft of the primary teeth and malocclusion is needed in the future.

### 4.1 Limitations of the study

There are certain constraints associated with this study. The effect of genetic factors on the prevalence of malocclusion was not included. Future studies should conduct oral examinations of the children and their immediate and collateral relatives and genetic analysis using gene sequencing to explore the role of genetic factors in the formation of malocclusion. In addition, this study did not include information on the bad oral habits of all children. Future studies should conduct separate one-on-one interviews with children and guardians to collect accurate information on children's bad habits. Finally, the trauma of primary anterior teeth was not counted in this survey because the permanent anterior teeth have erupted, and the future experimental design can design a cohort study from the age of 3 years to further clarify the effect of primary tooth trauma on malocclusion.

## 5 Conclusions

The present study provided information on the prevalence of malocclusion and its influencing factors in 7–8-year-old Zhuang children. Malocclusion was a common oral problem in Zhuang children; therefore, early attention should be paid to oral health. In addition to the current main concern about the effects of children's caries, the dangers of malocclusion in early childhood should also be emphasized, and the influencing factors of malocclusion should be prevented and intervened with. Therefore, this study provides a reference for future studies focusing on the prevention of malocclusion in children.

## Data Availability

The original contributions presented in the study are included in the article/Supplementary Material, further inquiries can be directed to the corresponding authors.
